# Renal protective effect of antiplatelet therapy in antiphospholipid antibody-positive lupus nephritis patients without antiphospholipid syndrome

**DOI:** 10.1371/journal.pone.0196172

**Published:** 2018-05-03

**Authors:** Hironari Hanaoka, Harunobu Iida, Tomofumi Kiyokawa, Yukiko Takakuwa, Takahiro Okazaki, Hidehiro Yamada, Shoichi Ozaki, Kimito Kawahata

**Affiliations:** 1 Division of Rheumatology and Allergology, Department of Internal Medicine, St. Marianna University School of Medicine, Kawasaki, Japan; 2 Medical Center for Rheumatic Diseases, Seirei Yokohama Hospital, Yokohama, Japan; Keio University, JAPAN

## Abstract

**Objective:**

We sought to evaluate the effect of antiplatelet therapy in addition to conventional immunosuppressive therapy for lupus nephritis (LN) patients positive for antiphospholipid antibodies (aPL) without definite antiphospholipid syndrome (APS).

**Methods:**

Patients with biopsy-proven LN class III or IV were retrospectively evaluated. We selected patients positive for anticardiolipin antibody (aCL) or lupus anticoagulant (LA) who did not meet the criteria for a diagnosis of APS. The patients were divided into two subgroups according to whether antiplatelet therapy was received. The cumulative complete renal response (CR) rate, relapse-free rate, and change in estimated glomerular filtration rate (eGFR) over 3 years after induction therapy were calculated.

**Results:**

We identified 17 patients who received antiplatelet therapy and 21 who did not. Baseline clinicopathological characteristics and immunosuppressive therapy did not show a significant difference between the two groups except for a higher incidence of LN class IV in the treatment group (p = 0.03). There was no difference in cumulative CR rate, relapse-free rate, or eGFR change between these subgroups. However, when data on LA-positive patients were assessed, an improvement in eGFR was found (p = 0.04) in patients receiving antiplatelet treatment.

**Conclusion:**

Addition of anti-platelet therapy was associated with an improvement of eGFR in LA-positive patients with LN class III or IV.

## Introduction

Lupus nephritis (LN) contributes to significant morbidity and mortality in systemic lupus erythematosus (SLE) [1, 2]. Antiphospholipid syndrome (APS) is characterized by a state of hypercoagulability which potentially affects all parts of the vascular system and can be associated with SLE [3]. APS is reported to worsen the prognosis of LN [4]. Based on its contribution to the renal outcome, the American College of Rheumatology (ACR) recently published recommendations for LN management [5], under which LN patients with APS should be treated with conventional immunosuppressive treatment plus antiplatelet or anticoagulation therapy. Although it has been reported that the presence of anticardiolipin antibodies (aCL) is a strong predictor of worse long-term renal outcome in LN regardless of whether the criteria for an APS diagnosis are met [6, 7], the renoprotective effect of antiplatelet therapy has not been evaluated.

Here, we analyzed the effect of adding antiplatelet agents to conventional immunosuppressive therapy for LN patients who were positive for aCL or lupus anticoagulant (LA) without definite APS.

## Materials and methods

### Patients

As described in detail previously [8], we performed a retrospective study of Japanese patients who met the ACR classification criteria for SLE [9] and who visited St. Marianna University Hospital from 2003 through 2010. All patients with biopsy-proven class III or IV LN according to the International Society of Nephrology/Renal Pathology Society (ISN/RPS) classification [10] were selected. Patients had to have received at least 3 years of care at the hospital. We selected patients who tested positive on two or more occasions at least 12 weeks apart for one of the following aPLs: aCL of IgG isotype, anti-β2 glycoprotein-I antibody of IgG isotype, or lupus anticoagulant (LA). The antibody titers were measured with a standard enzyme-linked immunosorbent assay (ELISA) [11, 12]. LA was tested according to the guidelines of the International Society on Thrombosis and Haemostasis (Scientific Subcommittee on LAs/phospholipid-dependent antibodies) [13, 14]. No patients fulfilled the criteria for a diagnosis of APS [15]. Of 358 SLE patients, 82 had biopsy-proven LN class III or IV. Two of these were lost to follow-up. Of the 80 remaining LN patients, 38 patients tested positive for one of the two antiphospholipid antibodies or LA as mentioned above, and their data were included. This study was approved by the Ethics Committee of St. Marianna University School of Medicine (approval number 3305). Since the study was conducted under a retrospective cohort design without any investigations/interventions done besides those for clinical use, written informed consent was not required. We retrospectively observed clinical course after induction therapy. This study was carried out as per routine clinical care and antiplatelet therapy was initiated at the attending physician’s discretion.

### Data collection

Clinical information was obtained from all records at baseline and at 2, 4, 8, 12, 24, 48, 96, and 144 weeks (3 years) after induction therapy. The baseline clinical information was collected at the time of renal biopsy before induction therapy. Data included demographic features, treatment regimens, and SLE disease activity index (SLEDAI) [16]. Complete renal response (CR) was defined based on the Joint European League Against Rheumatism and European Renal Association–European Dialysis and Transplant Association (EULAR/ERA-EDTA) guidelines for LN [5], with CR defined as a urine protein: creatinine ratio (UPCR) of 50 mg/mmol and normal or near-normal (within 10% of normal GFR if previously abnormal) renal function, substituting 0.5 g/g Cr for UPCR 50 mg/mmol [5]. Relapse was defined by nephritic and proteinuric flares according to EULAR/ERA-EDTA guidelines [5], with eGFR decreasing by ≥ 10%, active urine sediment, or increasing UPCR > 1.0 g/gCr after achieving CR. We also used the Systemic Lupus International Collaborating Clinics/American College of Rheumatology Damage Index (SDI) to define systemic damage accrual [17].

### Renal pathology

All patients underwent a renal biopsy before induction therapy. Specimens for light microscopy were embedded in paraffin, sectioned, and stained with Masson’s trichrome, hematoxylin–eosin, periodic acid silver–methenamine stain, and periodic acid–Schiff stain in all cases. Frozen tissue was cut into 5-μm sections and incubated with fluoresceinated antisera to human immunoglobulin (Ig) G, IgA, IgM, C3, C4, C1q, and fibrinogen. All patients were diagnosed according to the ISN/RPS classification [10] by light microscopy and immunofluorescence analysis. The activity index (AI) and the chronicity index (CI) [18] were calculated. Morphological features of the standard AI and CI were evaluated separately, namely endocapillary hypercellularity, polymorphonuclear leukocyte infiltration, karyorrhexis/fibrinoid necrosis, cellular crescents, hyaline deposits, interstitial inflammation, glomerular sclerosis, fibrous crescents, tubular atrophy, and interstitial fibrosis. The percentage of these features was measured in the individual patients.

### Statistical analysis

Continuous values are shown as mean ± standard deviation (SD). Differences between the groups were analyzed using the Mann-Whitney *U*-test or Kruskal-Wallis test for nonparametric data and the chi-squared or Fisher’s exact test for categorical data. Changes in eGFR from baseline to year 3 were analyzed using the Wilcoxon T-test. Cumulative CR rates and relapse-free rates were calculated using the Kaplan-Meier method, and differences between the two groups were tested with a log-rank test.

## Results

### Baseline clinicopathological characteristics and treatment regimens

The 38 cases were divided into two subgroups according to whether antiplatelet therapy was administered or not. Demographic and clinical features at baseline are shown in Table 1. Seventeen patients received antiplatelet treatment and 21 did not. Among clinicopathological features at baseline, patients who received antiplatelet therapy had a significantly higher incidence of LN class IV (p = 0.03). No patient had the pathological features of APS nephropathy (APSN) [15]. Although not significant, patients with treatment had a tendency to higher diastolic blood pressure (p = 0.09), higher titer of aCL-IgG (p = 0.09), and decreased tendency of LN class III and IV+V (p = 0.06 and p = 0.09, respectively). There were no remarkable differences between the two subgroups with regard to renal pathological findings, including morphological features of LN, or AI and CI.

**Table 1:** Baseline clinical and renal pathological features of antiphospholipid antibody-positive LN patients with or without antiplatelet therapy.

	Antiplatelet therapy		
Baseline Characteristics	Yes (n = 17)	No (n = 21)	p
Gender (% female)	17 (100.0)	17 (80.9)	0.06
Age (years)	38.3 ± 11.6	37.3 ± 8.9	0.41
BMI (kg/m^2^)	22.3 ± 4.9	22.3 ± 4.7	0.49
Systolic blood pressure (mmHg)	129.5 ± 13.6	127.3 ± 17.5	0.36
Diastolic blood pressure (mmHg)	82.1 ± 9.6	75.4 ± 13.7	0.09
HbA1c (%)	5.7 ± 0.3	5.6 ± 0.7	0.43
LDL-C (mg/dL)	123.1 ± 12.4	134.4 ± 11.8	0.33
Disease duration (years)	5.4 ± 7.0	8.6 ± 9.7	0.17
SLEDAI	16.1 ± 6.1	15.2 ± 5.9	0.34
SDI	0.5 ± 0.7	0.2 ± 0.6	0.11
Proteinuria (g/gCr)	2.9 ± 2.7	2.7 ± 2.6	0.40
eGFR (ml/min/1.73m^2^)	79.9 ± 36.4	81.8 ± 34.6	0.44
Anti-dsDNA antibody (IU/mL)	145.8 ± 147.6	207.4 ± 146.1	0.14
CH50 (U/ml)	14.3 ± 5.8	14.9 ± 8.1	0.41
aPL profile			
aCL-IgG positive (%)	8 (47.1)	9 (42.9)	0.79
titer (IU/mL)	49.8 ± 40.8	25.3 ± 24.6	0.09
β2GPI-IgG positive (%)	5 (29.4)	3 (14.3)	0.26
titer (U/mL)	46.5 ± 49.6	37.7 ± 58.2	0.40
LA positive (%)	11 (64.7)	10 (47.6)	0.29
Prednisolone (mg/day)	47.7 ± 16.0	42.0 ± 15.3	0.17
Induction therapy			
IVCY (%)	6 (35.3)	6 (28.6)	0.65
MMF (%)	6 (35.3)	5 (23.8)	0.43
Tacrolimus (%)	4 (23.5)	6 (28.6)	0.72
Others (%)	1 (5.8)	4 (19.0)	0.23
Renal pathological findings			
ISN/RPS classification			
III (A) or III (A/C) (%)	8 (47.1)	6 (28.6)	0.24
III (A) or III (A/C) + V (%)	0 (0)	4 (19.0)	0.06
IV (A) or IV (A/C) (%)	6 (35.3)	2 (9.5)	0.03
IV (A) or IV (A/C) + V (%)	3 (17.6)	9 (42.9)	0.09
Endocapillary hypercellularity (%)	40.1 ± 23.5	41.4 ± 33.1	0.34
Leukocyte infiltration (%)	3.9 ± 2.1	3.4 ± 1.3	0.29
Subendothelial hyaline deposits (%)	33.3 ± 29.6	31.8 ± 30.8	0.55
Fibrinoid necrosis/karyorrhexis (%)	6.4 ± 10.6	7.3 ± 8.1	0.48
Cellular crescents (%)	6.3 ± 10.3	7.6 ± 10.8	0.85
Interstitial inflammation (%)	5.2 ± 3.4	4.8 ± 6.0	0.46
Glomerular sclerosis (%)	3.0 ± 4.2	7.9 ± 9.2	0.10
Fibrous crescents (%)	4.6 ± 2.9	7.1 ± 4.7	0.10
Tubular atrophy (%)	4.8 ± 3.3	6.9 ± 4.1	0.09
Interstitial fibrosis (%)	4.8 ± 6.4	7.2 ± 3.9	0.09
Activity index	5.4 ± 2.9	6.9 ± 2.3	0.21
Chronicity index	1.0 ± 0.5	1.3 ± 1.5	0.32

SLEDAI, Systemic Lupus Erythematosus Disease Activity Index; SDI, Systemic Lupus International Collaborating Clinics/American College of Rheumatology Damage Index; dsDNA, double-stranded DNA; IVCY, intravenous cyclophosphamide; MMF, mycophenolate mofetil; aPL, antiphospholipid antibody; aCL, anticardiolipin antibody; β2GPI, anti-β2 glycoprotein-I antibody; LA, lupus anticoagulant.

Antiplatelet regimens, either low-dose aspirin (100 mg/day) or dipyridamole (300 mg/day), were initiated at the attending physician’s discretion after renal biopsy. All patients received induction therapy with glucocorticoids at an initial dose of 1.0 mg prednisolone equivalent/kg/day for 2–4 weeks. Glucocorticoids were then tapered by 10% of the last dose or 10 mg, as determined by the attending physician. Prednisolone dose did not differ markedly between the subgroups (p = 0.17) (Table 1). The dose of intravenous cyclophosphamide (IVCY) ranged from 500 mg every 2 weeks for 6 courses to 1000 mg every 4 weeks for 6 courses. Mycophenolate mofetil was started at an initial dose of 0.5–1.0 g/day and gradually increased to 2.0 g/day. Tacrolimus dose (1.5–3.0 mg/day) was precisely adjusted to a trough value of serum concentrations. After six infusions of IVCY, patients were switched to azathioprine 100 mg/day, while treatment with other immunosuppressants was continued as maintenance therapy.

### Cumulative CR rates and relapse-free rate

**Fig 1:**
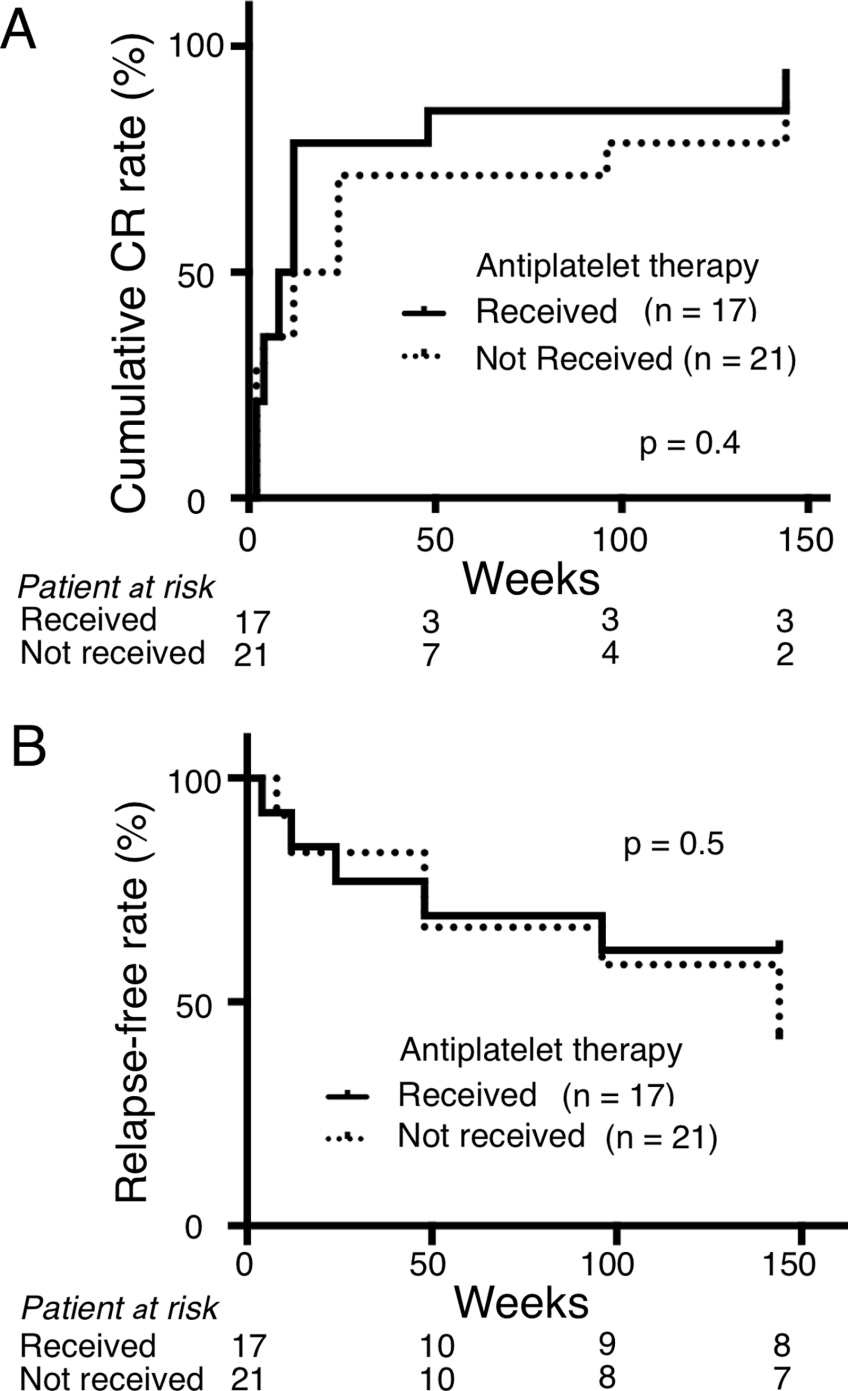
Effects of antiplatelet therapy on cumulative complete response rates or relapse-free rates. Cumulative complete renal response rate (A) and relapse-free rate (B) for 3 years after induction therapy depending on the antiplatelet treatment received. A full line indicates patients who received antiplatelet therapy and a dotted line indicates those who did not. There was no difference between these groups in terms of CR rate (p = 0.4) and relapse-free rate (p = 0.5). CR, Complete renal response.

Fig 1A shows cumulative CR rates of the two subgroups. Cumulative CR rates over 3 years were not different (p = 0.4). We further investigated the relapse-free rate over 3 years (Fig 1B), and found no differences between the subgroups (p = 0.5).

### Change in eGFR between baseline and year 3

**Fig 2:**
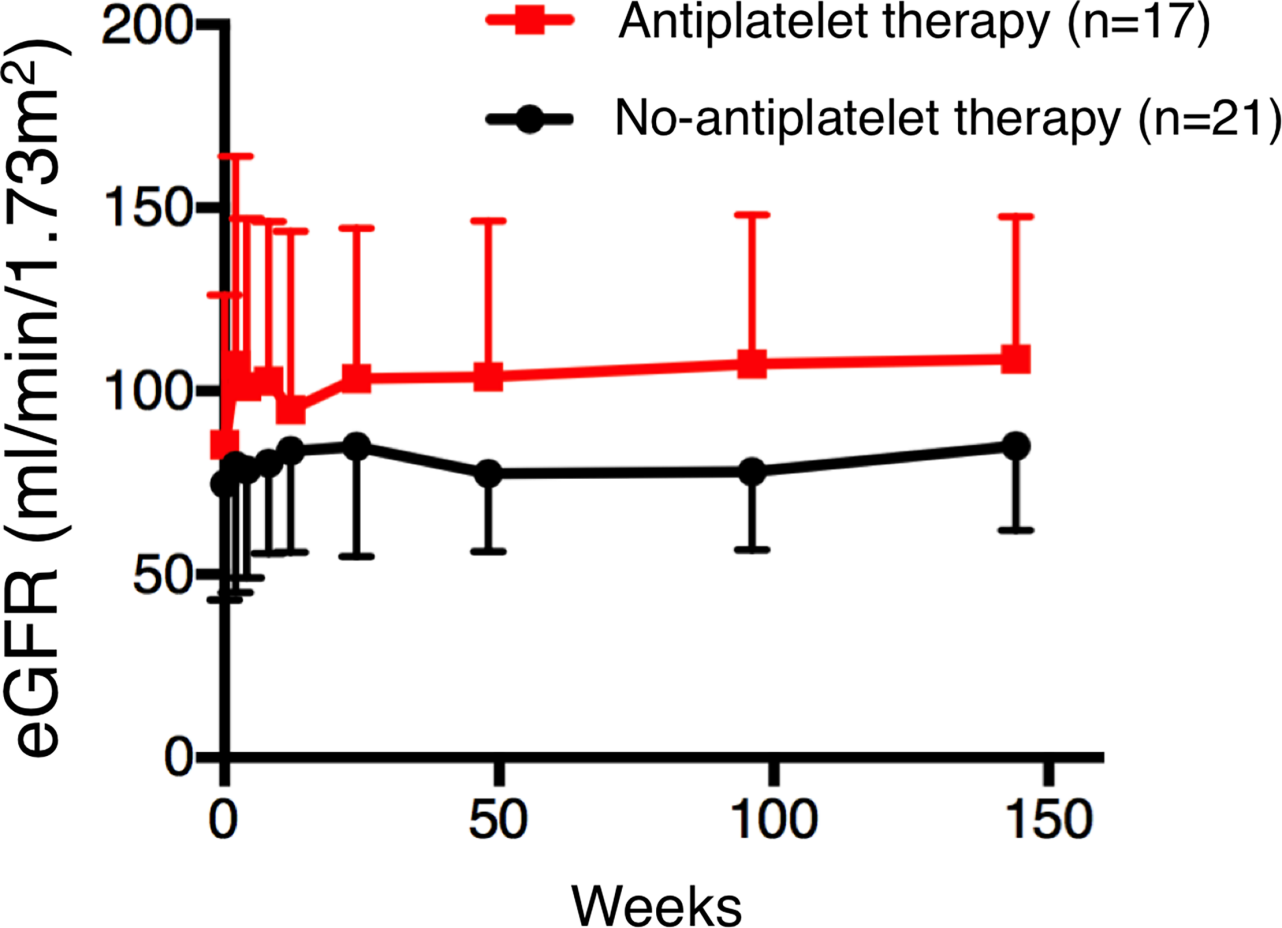
Change in eGFR levels over 3 years. There was no difference in eGFR level between patients with antiplatelet therapy and those without at any observational points. eGFR, estimated glomerular filtration rate.

We analyzed changes in eGFR over 3 years after induction therapy in the two subgroups (Fig 2), and found no difference at any observational points. Since the highest eGFR level was found at year 3 in both groups, we next compared the difference between baseline and year 3 with or without antiplatelet treatment for LA positivity.

### Analysis according to LA positivity

**Fig 3:**
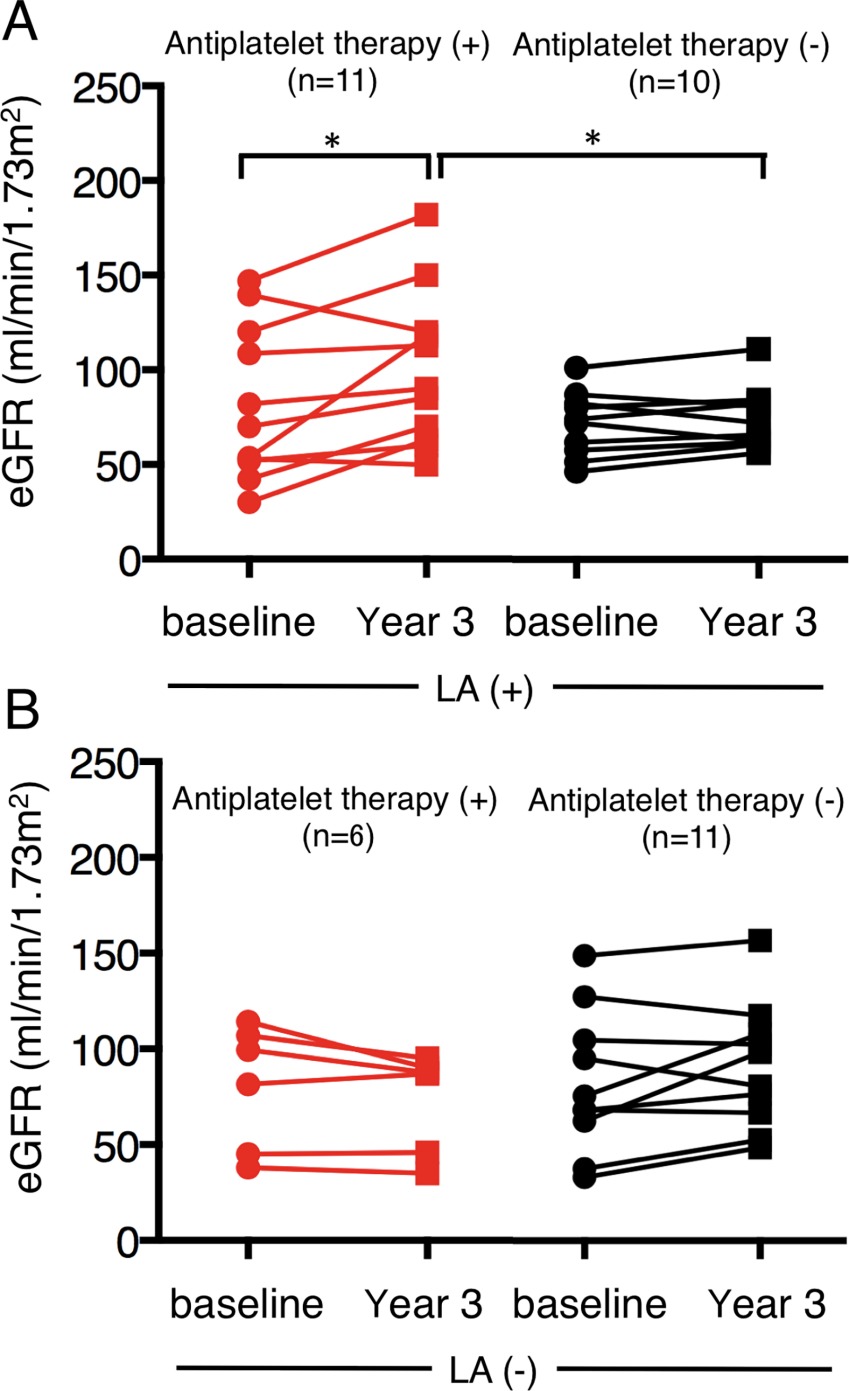
Change in eGFR levels between baseline and year 3 depending on LA positivity. (A) Change in eGFR in LA-positive patients. Patients given antiplatelet therapy had an improvement in eGFR from baseline to year 3 compared to those not given therapy (p = 0.04). The level of eGFR at year 3 was significantly higher in LA-positive patients receiving antiplatelet therapy (p = 0.04). (B) Change in eGFR in LA-negative patients. No improvement was seen. eGFR, estimated glomerular filtration rate; LA, lupus anticoagulant. *, p = 0.04.

We divided all the patients into two different subgroups depending on LA positivity and the same analysis was performed. Table 2 shows baseline clinicopathological features according to LA status. A significantly lower incidence of female sex was observed in the LA-negative subgroup not treated with antiplatelet therapy (p = 0.01). The LA positive/antiplatelet treatment group was younger, and had a higher eGFR level and lower chronicity index, although not significant. We found no significant difference in cumulative CR rate and relapse-free rate depending on the LA positivity. Fig 3 shows the change in eGFR between baseline and year 3 according to LA status. We found an improvement in the LA-positive subgroup given antiplatelet therapy (p = 0.04). These patients had a higher eGFR level at year 3 than the LA-positive subgroup which did not receive antiplatelet therapy (p = 0.04). We found no significant differences when we conducted the same analysis according to aCL or β2GPI-IgG positivity.

**Table 2:** Baseline clinical and renal pathological features according to LA status.

	LA-positive		LA-negative		
Baseline characteristic	Antiplatelet therapy (n = 11)	No antiplatelet therapy (n = 10)	Antiplatelet therapy (n = 6)	No antiplatelet therapy (n = 11)	p
Gender (% female)	11 (100.0)	10 (100.0)	6 (100.0)	7 (63.6)	0.01
Age (years)	36.2 ± 12.6	42.8 ± 13.2	40.2 ± 11.1	35.4 ± 6.6	0.56
BMI (kg/m^2^)	21.0 ± 2.7	21.6 ± 4.2	24.0 ± 7.0	22.5 ± 5.0	0.74
Systolic blood pressure (mmHg)	124.7 ± 13.5	141.2 ± 19.3	135.4 ± 12.7	121.2 ± 14.1	0.11
Diastolic blood pressure (mmHg)	78.8 ± 5.1	83.0 ± 21.2	86.0 ± 12.7	72.3 ± 9.2	0.17
HbA1c (%)	5.8 ± 0.2	5.4 ± 0.5	5.3 ± 0.7	5.9 ± 0.1	0.34
LDL-C (mg/dL)	128.4 ± 15.0	133.1 ± 20.3	126.5 ± 12.4	135.2 ± 14.3	0.33
Disease duration (years)	4.5 ± 6.9	7.7 ± 12.2	6.6 ± 7.5	9.0 ± 9.3	0.77
SLEDAI	17.3 ± 6.4	18.0 ± 2.4	14.7 ± 3.7	14.1 ± 6.5	0.53
SDI	0.3 ± 0.4	0.3 ± 0.5	0.7 ± 0.8	0.2 ± 0.4	0.38
Proteinuria (g/gCr)	3.2 ± 3.5	2.9 ± 2.2	2.5 ± 1.3	2.5 ± 2.8	0.96
eGFR (mL/min/1.73m^2^)	80.3 ± 34.7	71.1 ± 24.6	75.6 ± 41.8	85.3 ± 37.8	0.92
Anti-dsDNA antibody (IU/mL)	177.6 ± 172.7	183.0 ± 156.8	108.6 ± 115.7	216.3 ± 149.3	0.58
CH50 (U/mL)	14.0 ± 4.6	13.6 ± 4.2	14.6 ± 7.2	15.4 ± 9.2	0.87
aPL profile					
aCL-IgG positive (%)	5 (45.5)	3 (30.0)	3 (50.0)	6 (54.5)	0.77
titer (IU/mL)	51.3 ± 40.8	50.5 ± 48.0	41.9 ± 42.7	18.4 ± 10.9	0.19
β2GPI-IgG positive (%)	3 (27.3)	2 (20.0)	2 (33.3)	1 (9.1)	0.62
titer (U/mL)	73.5 ± 27.5	67.5 ± 29.2	19.4 ± 3.4	5.4 ± 1.1	0.40
Prednisolone (mg/day)	43.6 ± 10.3	46.3 ± 7.5	52.5 ± 20.9	40.5 ± 17.4	0.52
Remission induction therapy					
IVCY (%)	3 (27.2)	2 (20.0)	3 (50.0)	4 (36.4)	0.67
MMF (%)	4 (36.4)	3 (30.0)	2 (33.3)	2 (18.2)	0.81
TAC (%)	3 (27.3)	3 (30.0)	1 (16.7)	3 (27.3)	0.95
Others (%)	1 (9.1)	2 (20.0)	0 (0.0)	2 (18.2)	0.51
Renal pathological findings					
ISN/RPS classification					
III (A) or III (A/C) (%)	6 (54.5)	3 (30.0)	2 (33.3)	3 (27.3)	0.43
III (A) or III (A/C) + V (%)	0 (0.0)	2 (20.0)	0 (0.0)	2 (18.2)	0.28
IV (A) or IV (A/C) (%)	3 (27.3)	1 (10.0)	3 (50.0)	1 (9.1)	0.30
IV (A) or IV (A/C) + V (%)	2 (18.2)	4 (40.0)	1 (16.7)	5 (45.5)	0.20
Endocapillary hypercellularity (%)	34.2 ± 30.1	38.9 ± 31.5	46.2± 10.4	40.6 ± 19.2	0.34
Leukocyte infiltration (%)	3.0 ± 3.1	3.5 ± 4.8	4.0 ± 6.4	3.4 ± 5.1	0.67
Subendothelial hyaline deposits (%)	19.0 ± 13.4	34.6 ± 29.3	40.0 ± 29.1	30.0 ± 26.1	0.12
Fibrinoid necrosis/karyorrhexis (%)	5.1 ± 10.6	7.1 ± 2.9	7.3 ± 6.4	6.0 ± 3.2	0.18
Cellular crescents (%)	5.8 ± 9.2	7.6 ± 3.1	7.2 ± 6.1	7.1 ± 2.1	0.28
Interstitial inflammation (%)	5.0 ± 2.1	3.9 ± 8.0	5.4 ± 3.1	4.9 ± 4.6	0.66
Glomerular sclerosis (%)	2.0 ± 1.8	6.9 ± 7.9	5.0 ± 4.8	8.0 ± 7.4	0.15
Fibrous crescents (%)	2.6 ± 3.4	8.9 ± 2.1	4.9 ± 11.1	6.3 ± 1.6	0.10
Tubular atrophy (%)	2.1 ± 3.2	6.7 ± 6.9	5.3 ± 2.2	7.0 ± 3.1	0.26
Interstitial fibrosis (%)	2.7 ± 6.8	7.3 ± 4.2	5.3 ± 3.9	7.1 ± 3.8	0.32
Activity index	4.7 ± 3.3	5.9 ± 2.3	5.5 ± 0.2	7.1 ± 3.6	0.66
Chronicity index	0.9 ± 1.8	1.3 ± 1.2	1.2 ± 1.5	1.4 ± 1.3	0.43

SLEDAI, Systemic Lupus Erythematosus Disease Activity Index; SDI, Systemic Lupus International Collaborating Clinics/American College of Rheumatology Damage Index; dsDNA, double-stranded DNA; IVCY, intravenous cyclophosphamide; ELNT, Euro-lupus nephritis trial; MMF, mycophenolate mofetil; TAC, tacrolimus; aPL, antiphospholipid antibody; aCL, anticardiolipin antibody; β2GPI, anti-β2 glycoprotein-I antibody; LA, lupus anticoagulant, HbA1c, hemoglobin A1c; LDL-C, low-density lipoprotein cholesterol.

## Discussion

In this study, we found that antiplatelet therapy in addition to conventional immunosuppressive therapy was associated with improvement of eGFRs in LA-positive patients over 3 years. LA-negative patients experienced no such improvement.

Prophylaxis strategies in asymptomatic aPL-positive SLE patients are poorly investigated. Although the randomized controlled clinical trial of primary thrombosis prevention in asymptomatic, persistently aPL-positive individual without the definite APS has been conducted, these individuals do not benefit from aspirin for primary thrombosis prophylaxis [19]. However, this study did not include SLE patients and only investigated cumulative thrombosis incidence rate. Renal protective effect of anti-platelet therapy for aPL-positive LN patients without any history of thrombotic events is still controversial.

In our study of patients with LN, a benefit from antiplatelet therapy was only seen in the LA-positive patients. Although the relationship between the subtype of aPL and the development of APSN is unclear, some studies have found a link between LA, but not aCL, and APSN in SLE patients [20–22]. It has been suggested that APS may develop in up to 50% of patients with SLE serologically positive for aPL without thrombotic events after 20 years of follow-up [23]. Our investigations suggest that LA-positive patients, who are at risk for progression to APS or APSN, stand to benefit from antiplatelet treatment. However, it has been reported that some patients who initially test positive for aCL subsequently test negative for those antibodies over long-term observation [24]. Since we did not evaluate sustained seropositivity for all patients, this may make our findings less convincing.

Although we found a beneficial effect of antiplatelet therapy in LA-positive patients in our study, it is still controversial whether antiplatelet agents or anticoagulants are more beneficial. One report has described the prevention of thrombotic recurrence in the kidney using anticoagulation in APSN [25], but the role of anticoagulation in the preservation of renal function is still unknown. Daugas et al. reported a significant association of APSN with LA and extrarenal APS, mainly manifested by arterial thrombosis [20]. This report supports the use of antiplatelet therapy for APSN. We did not evaluate the effects of anticoagulation and solving this issue will clearly require additional studies.

This study was conducted single-center, retrospective design, very small number and only Japanese population was assessed. As patients were selected retrospectively, selection bias was present as follows; addition of antiplatelet therapy was decided by the attending physicians and only the patients who could be observed for 3 years were selected. Attending physicians may decide the treatment regimen based on lupus manifestation, laboratory data including APS test, and renal pathology. We found combination with class V was found more in no-antiplatelet group than antiplatelet therapy group. Since mixed type (+V) had poor renal outcome [26], this pathological difference may influence the result. Furthermore, we could not measure aCL-IgM or β2GPI-IgM, which underestimated the disease population and we combined low-dose aspirin and dipyridamole as an antiplatelet therapy to evaluate its efficacy, which may be separately evaluated. Therefore, a multi-center, prospective study is required to confirm our findings.

In conclusion, we found that antiplatelet therapy in addition to conventional immunosuppressive therapy was associated with improvement of eGFR in LA-positive LN patients not meeting the criteria for a diagnosis of APS. This suggests that there may be a wider indication for antiplatelet therapy in LN, in addition to its use in patients with a definite APS diagnosis.

## Supporting information

S1 Table CR achievement in patients with antiplatelet therapy and those without. (PDF)

S2 Table Flare in patients with antiplatelet therapy and those without. (PDF)

S3 Table Change of eGFR in patients with antiplatelet therapy and those without. (PDF)
