# Supplementary material for: Renal protective effect of antiplatelet therapy in antiphospholipid antibody-positive lupus nephritis patients without antiphospholipid syndrome
Source: PLoS One. 2018 May 3;13(5):e0196172. doi: 10.1371/journal.pone.0196172 (PMC5933765; doi:10.1371/journal.pone.0196172)
Supplement: S1 Table — (PDF) [file pone.0196172.s001.pdf]

Patients without antiplatelet therapy

No	Weeks	CR
1	144	0
2	4	1
3	12	1
4	2	1
5	96	1
6	24	1
7	144	0
8	2	1
9	144	1
10	24	1
11	2	1
12	24	1
13	12	1
14	2	1
15	144	0
16	2	1
17	144	1
18	24	1
19	2	1
20	24	1
21	12	1

Patients with antiplatelet therapy

No	Weeks	CR
1	8	1
2	12	1
3	144	1
4	2	1
5	144	0
6	4	1
7	12	1
8	12	1
9	8	1
10	2	1
11	12	1
12	2	1
13	48	1
14	8	1
15	12	1
16	144	1
17	4	1
