# Supplementary material for: Renal protective effect of antiplatelet therapy in antiphospholipid antibody-positive lupus nephritis patients without antiphospholipid syndrome
Source: PLoS One. 2018 May 3;13(5):e0196172. doi: 10.1371/journal.pone.0196172 (PMC5933765; doi:10.1371/journal.pone.0196172)
Supplement: S2 Table — (PDF) [file pone.0196172.s002.pdf]

Patients without antiplatelet therapy

No	Weeks	Flare
1	144	1
2	8	1
3	144	1
4	96	1
5	144	0
6	144	0
7	144	0
8	144	0
9	48	1
10	48	1
11	144	0
12	12	1
13	144	1
14	8	1
15	144	1
16	96	1
17	144	0
18	144	0
19	144	0
20	144	0
21	144	0

Patients with antiplatelet therapy

No	Weeks	Flare
1	96	1
2	48	1
3	144	0
4	144	0
5	144	0
6	144	0
7	144	0
8	12	1
9	144	0
10	96	1
11	48	1
12	144	0
13	144	0
14	24	1
15	144	0
16	144	0
17	4	1
