# Supplementary material for: Renal protective effect of antiplatelet therapy in antiphospholipid antibody-positive lupus nephritis patients without antiphospholipid syndrome
Source: PLoS One. 2018 May 3;13(5):e0196172. doi: 10.1371/journal.pone.0196172 (PMC5933765; doi:10.1371/journal.pone.0196172)
Supplement: S3 Table — (PDF) [file pone.0196172.s003.pdf]

Change of eGFR for 144 weeks

Patients without antiplatelet therapy

	Week								
No	0	2	4	8	12	24	48	96	144
1	53.0	58.6	60.2	57.1	49.9	60.2	53.0	57.9	55.0
2	97.2	97.2	85.5	97.2	97.2	97.2	85.5	81.5	82.4
3	33.8	37.9	36.9	40.9	46.9	58.8	51.6	42.4	60.0
4	152.1	153.7	143.7	81.2	83.1	84.1	77.7	78.3	85.5
5	38.5	38.5	41.8	33.3	35.7	35.7	45.2	52.8	53.9
6	98.4	120.1	120.1	120.1	120.1	153.4	118.8	96.3	116.4
7	69.6	61.2	61.2	80.2	80.6	95.4	79.8	79.0	88.0
8	84.7	59.5	52.3	89.7	76.0	80.1	74.7	66.8	78.7
9	77.2	68.8	87.9	101.7	129.8	98.6	89.1	92.9	110.3
10	56.2	106.8	83.1	90.3	106.8	90.2	85.4	110.5	110.5
11	69.9	78.5	69.9	78.5	78.5	78.5	69.4	68.8	90.0
12	45.2	50.7	66.7	70.3	96.4	50.7	62.8	62.8	54.6
13	106.9	78.3	87.6	90.1	88.0	106.9	106.2	105.4	104.7
14	63.9	105.6	102.2	97.4	83.0	98.9	87.7	97.4	100.5
15	74.8	79.7	78.5	80.6	83.7	84.9	77.6	78.1	85.0
16	76.3	80.1	79.3	80.3	84.1	85.3	77.7	79.0	86.2
17	74.0	81.2	80.3	78.2	83.2	84.8	77.0	79.1	84.2
18	77.1	79.5	80.1	81.3	83.2	85.3	77.4	78.5	85.2
19	73.2	79.4	80.3	78.2	82.9	83.7	76.8	78.3	85.7
20	74.1	79.8	78.4	79.7	83.8	84.9	77.9	78.5	85.8
21	73.8	80.2	77.9	80.4	84.2	86.0	78.1	78.5	85.0

Patients with antiplatelet therapy

	Week								
No	0	2	4	8	12	24	48	96	144
1	82.0	119.7	98.4	105.3	87.0	115.2	103.5	111.0	119.7
2	143.8	116.1	132.4	114.1	118.2	140.7	135.0	140.7	110.3
3	108.6	143.3	158.7	127.7	97.5	100.9	100.9	102.8	112.8
4	29.6	46.1	51.8	68.1	58.9	51.8	52.4	59.7	56.6
5	104.3	115.2	70.0	129.5	126.9	119.6	135.1	98.6	98.6
6	99.5	86.3	76.1	61.4	58.2	68.0	73.2	69.7	87.6
7	42.3	33.0	28.9	42.3	40.3	46.4	62.1	72.6	70.1
8	139.6	230.6	173.1	188.9	207.8	169.5	159.6	145.3	162.8
9	22.3	34.9	37.5	42.3	39.9	49.3	27.3	30.0	37.7
10	83.3	84.1	94.3	97.2	107.1	110.8	123.6	136.7	131.1
11	53.7	100.3	120.0	124.4	62.7	113.9	110.2	120.0	117.9
12	114.2	161.6	118.0	145.0	120.0	145.0	148.1	158.0	148.1
13	107.0	129.1	126.7	116.3	142.0	129.1	150.9	142.0	150.9
14	86.9	107.7	98.9	104.8	97.4	104.6	106.3	106.7	108.0
15	88.2	102.9	99.1	103.8	98.1	103.2	107.1	109.1	107.8
16	87.6	101.3	100.2	102.9	98.4	102.1	105.1	105.2	100.6
17	88.1	106.9	99.1	103.9	98.1	103.8	105.5	104.9	103.2
